# Exceptional response to furmonertinib in lung adenocarcinoma harboring HER2 exon 20 insertion mutation: a case report

**DOI:** 10.3389/fonc.2026.1749154

**Published:** 2026-02-26

**Authors:** Lingyun Lou, Junwei Tu, Zhuoyang Zhao, Saibin Wang

**Affiliations:** 1 Department of Pulmonary and Critical Care Medicine, Jinhua Municipal Central Hospital, Jinhua, Zhejiang, China; 2 Department of Pathology, Jinhua Municipal Central Hospital, Jinhua, Zhejiang, China; 3 School of Medicine, Shaoxing University, Shaoxing, Zhejiang, China

**Keywords:** case report, exon 20 insertion, furmonertinib, HER2, non-small cell lung cancer

## Abstract

**Background:**

The management of human epidermal growth factor receptor 2 (HER2)-mutant non-small cell lung cancer (NSCLC) remains a significant clinical challenge, with limited effective and accessible treatment options beyond antibody-drug conjugates such as trastuzumab deruxtecan (T-DXd). Furmonertinib, a third-generation EGFR tyrosine kinase inhibitor (TKI) with enhanced hydrophobic properties due to its trifluoroethoxy group, has shown activity against EGFR exon 20 insertions (ex20ins) but has not been explored in HER2-mutant NSCLC.

**Case description:**

A 65-year-old male smoker presented with progressive dyspnea and a performance status (PS) of 2. Initial computed tomography (CT) in March 2025 revealed bilateral pneumonic infiltrates. Biopsy confirmed T4N0M1 lung adenocarcinoma harboring the “ERBB2 p.Y772_A775dup” mutation. Administration of furmonertinib at a double standard dose of 160 mg/day resulted in symptomatic improvement and early radiological improvement within 5 days. Following chemotherapy and sintilimab failure in August 2025 due to progressive disease, furmonertinib rechallenge at 160 mg/day again induced a response within 5 days, with no grade ≥3 adverse events.

**Conclusion:**

This case provides the first clinical evidence of furmonertinib’s activity against HER2 ex20ins mutations. The structural homology between HER2 p.Y772_A775dup and EGFR exon 20 “near-loop” insertions may facilitate TKI binding. Furmonertinib emerges as a potential, cost-effective oral therapeutic alternative for this patient population, especially when standard therapies are not feasible, warranting further prospective investigation.

## Background

Non-small cell lung cancer (NSCLC) accounts for approximately 85% of lung cancer cases and is a leading cause of cancer-related mortality worldwide (1). Various human epidermal growth factor receptor 2 (HER2) alterations have been identified in NSCLC, including protein overexpression (2-35%), gene amplification (2-20%), and gene mutations (1-4%) (2, 3). HER2 mutations are currently a major focus of research in lung cancer, with the most common mutations occurring in exon 20, such as A775_G776insYVMA (also known as p.Y772_A775dup), which is the most frequent (4, 5). HER2-mutant NSCLC is characterized by aggressive features, including rapid progression and poor prognosis. Current therapies include platinum-based chemotherapy, HER2-targeted tyrosine kinase inhibitors (TKIs), monoclonal antibodies, and antibody-drug conjugates (ADCs) (3).

Furmonertinib is a novel third-generation epidermal growth factor receptor (EGFR)-TKI featuring a trifluoroethoxy group, which confers strong hydrophobicity, high lipophilicity, and significant electron-withdrawing properties (6). Compared to other third-generation EGFR-TKIs, furmonertinib has demonstrated good efficacy and tolerability against EGFR exon 20 insertion (ex20ins) mutant lung adenocarcinoma (7–9). However, its activity against HER2 ex20ins remains unexplored.

Here, we report for the first time a case of a patient with lung adenocarcinoma harboring a HER2 ex20ins mutation who responded to furmonertinib treatment.

## Case description

### Clinical presentation

**Figure 1:**
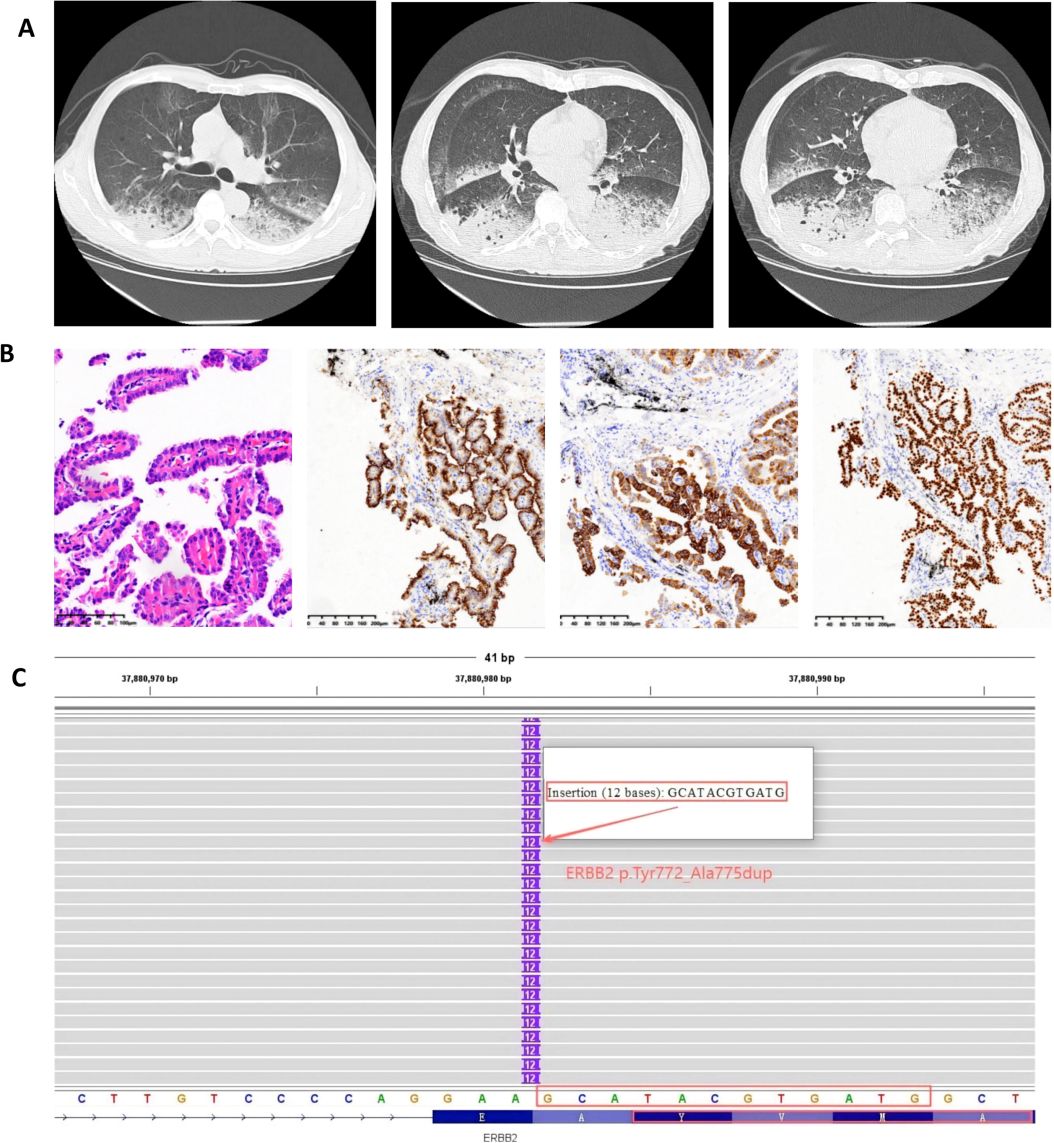
Imaging, histopathology and molecular diagnostics. **(A)** Baseline CT imaging, **(B)** Histopathology (200×) and Immunohistochemistry (100×): Adenocarcinoma Markers CK7(+), Napsin A(+), TTF-1(+), **(C)** Pre-therapy NGS of Lung Adenocarcinoma showing ERBB2 p.Y772_A775dup (VAF 18.58%).

A 65-year-old male (height 176 cm, weight 68 kg) was admitted on March 11, 2025, due to “cough with sputum, chest tightness, and shortness of breath for 4 months, worsening over 3 days.” He had no history of hypertension or diabetes but was a smoker with a 40-pack-year history (20 cigarettes/day). Physical examination revealed a performance status (PS) score of 1-2. No palpable lymphadenopathy was detected in the cervical, supraclavicular, or axillary regions. Cardiopulmonary examination was unremarkable, with normal heart sounds and clear lung fields on auscultation. After admission, he initially presented with pneumonia-like symptoms unresponsive to antibiotic therapy. A chest computed tomography (CT) on March 17 showed multiple inflammatory lesions in both lungs and pulmonary emphysema (**Figure 1A**). To establish a diagnosis, bronchoscopy under local anesthesia was performed. Subsequent to the pathological diagnosis of papillary lung adenocarcinoma (cT4N0M1, stage IVa) from a biopsy of the left lower lobe basal segment (**Figure 1B**), molecular analysis of the tumor was initiated.

### Treatment

**Figure 2:**
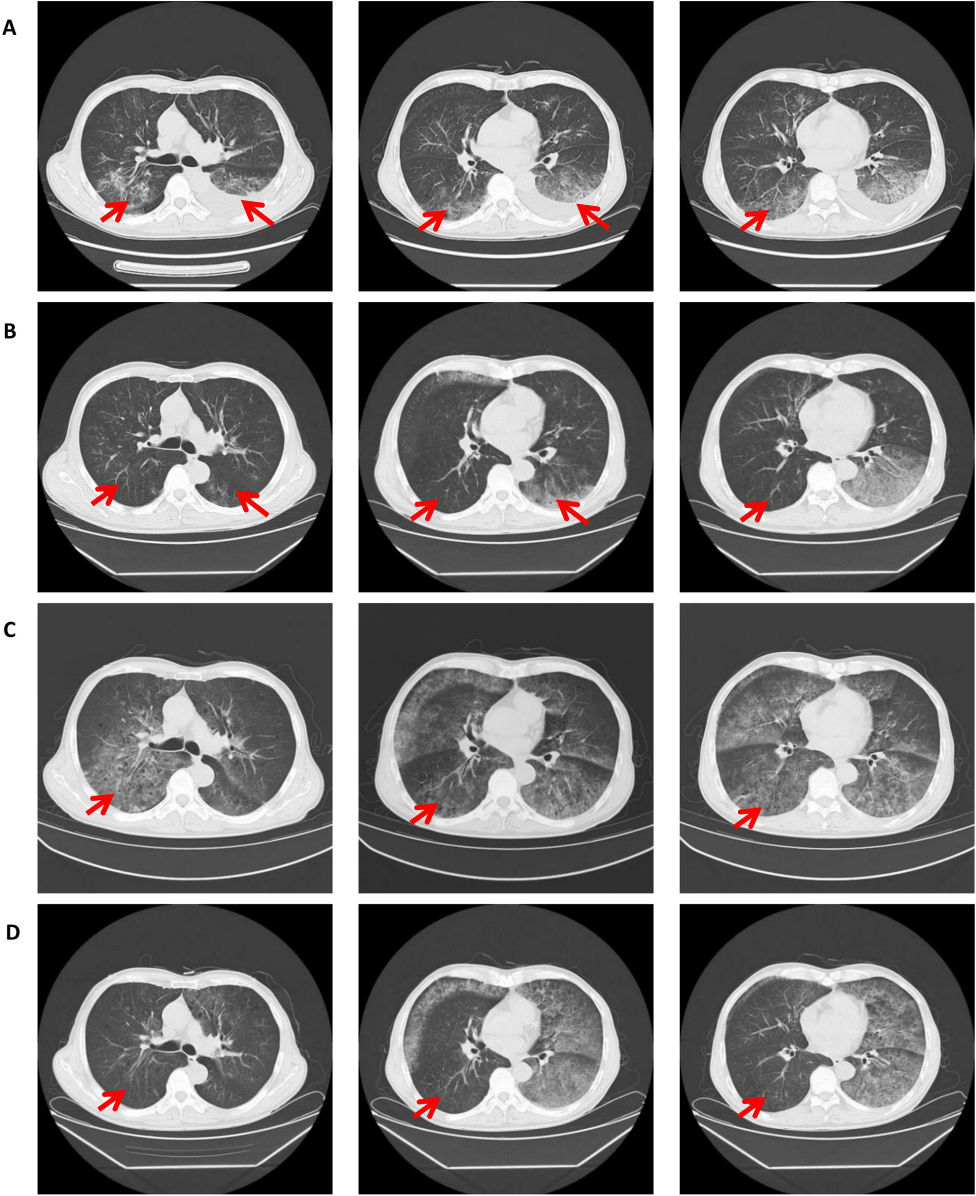
Serial computed tomography (CT) imaging of the chest demonstrates the dynamic radiographic changes in response to sequential therapies. **(A)** Scan after 5 days of furmonertinib therapy shows early radiographic improvement. **(B)** Following 45 days of continuous furmonertinib, a significant radiographic improvement is evident. **(C)** Subsequent imaging after 2 cycles of platinum-based chemotherapy combined with sintilimab demonstrated disease progression. **(D)** Re-challenge with furmonertinib for 5 days shows early radiographic improvement.

**Figure 3:**
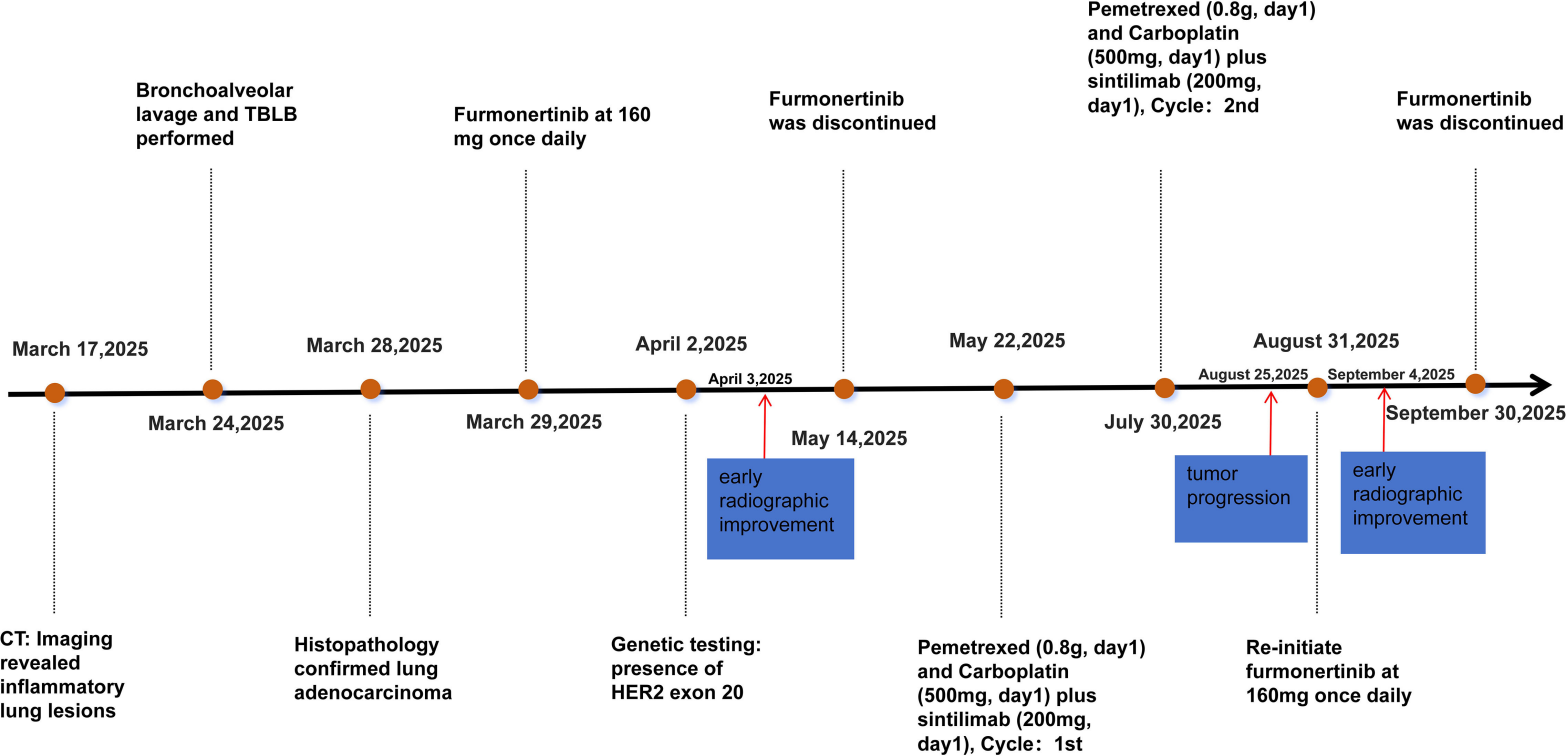
Timeline of the patient’s events from diagnosis to treatment.

As the disease progressed, the patient required high-flow nasal oxygen(HFNC), had a PS of 3-4, and was in poor general condition, unable to tolerate chemotherapy. After thorough discussion between the treating physician and the patient’s family, and considering the patient’s critical condition, financial constraints, and the lack of effective standard options, furmonertinib was initiated at a dose of 160 mg orally once daily. The patient and family provided informed consent for this off-label, high-dose use. The patient’s chest tightness improved markedly. A repeat chest CT after 5 days of treatment showed early radiological improvement in the lung lesions (**Figure 2A**). Subsequent next-generation sequencing (NGS) confirmed a clonal HER2 exon 20 insertion mutation (ERBB2 p.Y772_A775dup; VAF 18.58%), with no concurrent driver mutations or HER2 amplification detected (**Figure 1C**). Tumor mutational burden (TMB) was not assessed. Upon achieving a performance status of 1 with stable disease, furmonertinib was discontinued on May 14, 2025, to allow for the initiation of standard first-line chemotherapy. Between May 23 and July 30, 2025, the patient received two cycles of pemetrexed (0.8 g, day 1) and carboplatin (500 mg, day 1) plus sintilimab (200 mg, day 1). On August 25, the patient experienced significant shortness of breath and chest tightness upon minimal exertion. A repeat chest CT indicated clear tumor progression (**Figure 2C**). The National Comprehensive Cancer Network (NCCN) guidelines recommend T-DXd for patients with HER2-mutant NSCLC who have previously received first-line standard therapy, but the patient opted against it due to cost. After extensive discussion, considering the patient’s financial constraints and previous response to furmonertinib, the family decided to re-initiate furmonertinib at 160 mg once daily. A repeat chest CT after 5 days of furmonertinib treatment showed improvement compared to the previous scan (**Figure 2D**), accompanied by alleviation of chest tightness and shortness of breath. The patient was discharged on furmonertinib following clinical improvement. The timeline is shown in **Figure 3**.

### Follow-up and safety

The patient continued furmonertinib for approximately one month before discontinuing due to financial constraints. A follow-up chest CT on December 28, 2025, showed stable disease compared to previous imaging. Thereafter, the patient declined further chemotherapy, immunotherapy, or other antitumor treatments. Given the natural history of advanced NSCLC and the cessation of all active therapy, disease progression is anticipated. During both treatment periods with furmonertinib, no treatment-related adverse events ≥ grade 3 were reported. Telephone follow-up at home also indicated no apparent side effects.

## Discussion

The patient in this case is an elderly male who presented with lung adenocarcinoma manifesting as pneumonia, with clinical symptoms including copious sputum and significant chest tightness and shortness of breath. At the time of diagnosis, his condition was critical. As the genetic test report was pending and the patient was unable to tolerate chemotherapy, furmonertinib was initiated at 160 mg once daily, following discussion with his family. His symptoms improved, and he was discharged. The subsequent genetic test result identified an ERBB2 exon 20 mutation. He subsequently received two cycles of chemotherapy combined with immunotherapy, but the efficacy was suboptimal. Given disease progression and cost considerations, the decision was made to resume furmonertinib, which led to a positive clinical response.

For HER2-mutant NSCLC patients, the current first-line standard treatment is platinum-based chemotherapy with or without immune checkpoint inhibitors. However, the overall response rate (ORR) to platinum-based chemotherapy is approximately 43%, with a median progression-free survival (mPFS) of around 6 months (10). Nevertheless, the combination of chemotherapy and immunotherapy yields a mPFS of 8.5 months, a result that remains suboptimal (11). The poor response to chemotherapy and immunotherapy in this case aligns with previous study findings (10–12). Other treatment modalities include monoclonal antibodies, such as trastuzumab and pertuzumab, but their limited efficacy may be due to inadequate tumor penetration and insufficient blockade of downstream signaling. Tyrosine kinase inhibitors, including pan-HER TKIs (e.g., dacomitinib, neratinib, afatinib), EGFR/HER2 TKIs (e.g., lapatinib, pyrotinib, mobocertinib, BAY2927088), and selective HER2 TKIs (e.g., tucatinib, zongertinib), have yielded disappointing results in studies, with ORRs ranging from 0% to 42% and mPFS from 2.8 to 5.6 months (3). Among these, pyrotinib has shown the most promising activity, although its tolerability is limited by diarrhea, anemia, and rash. Novel therapeutic agents include anti-HER2 ADCs such as T-DXd and ado-trastuzumab emtansine (T-DM1), which have also been investigated for treating NSCLC patients with HER2 mutations. T-DXd is the first FDA-approved targeted therapy for previously treated HER2-mutant NSCLC at a dose of 5.4 mg/kg (13). While these drugs demonstrate clinical benefit, their widespread use is limited by cost and treatment-related side effects (14).

**Figure 4:**
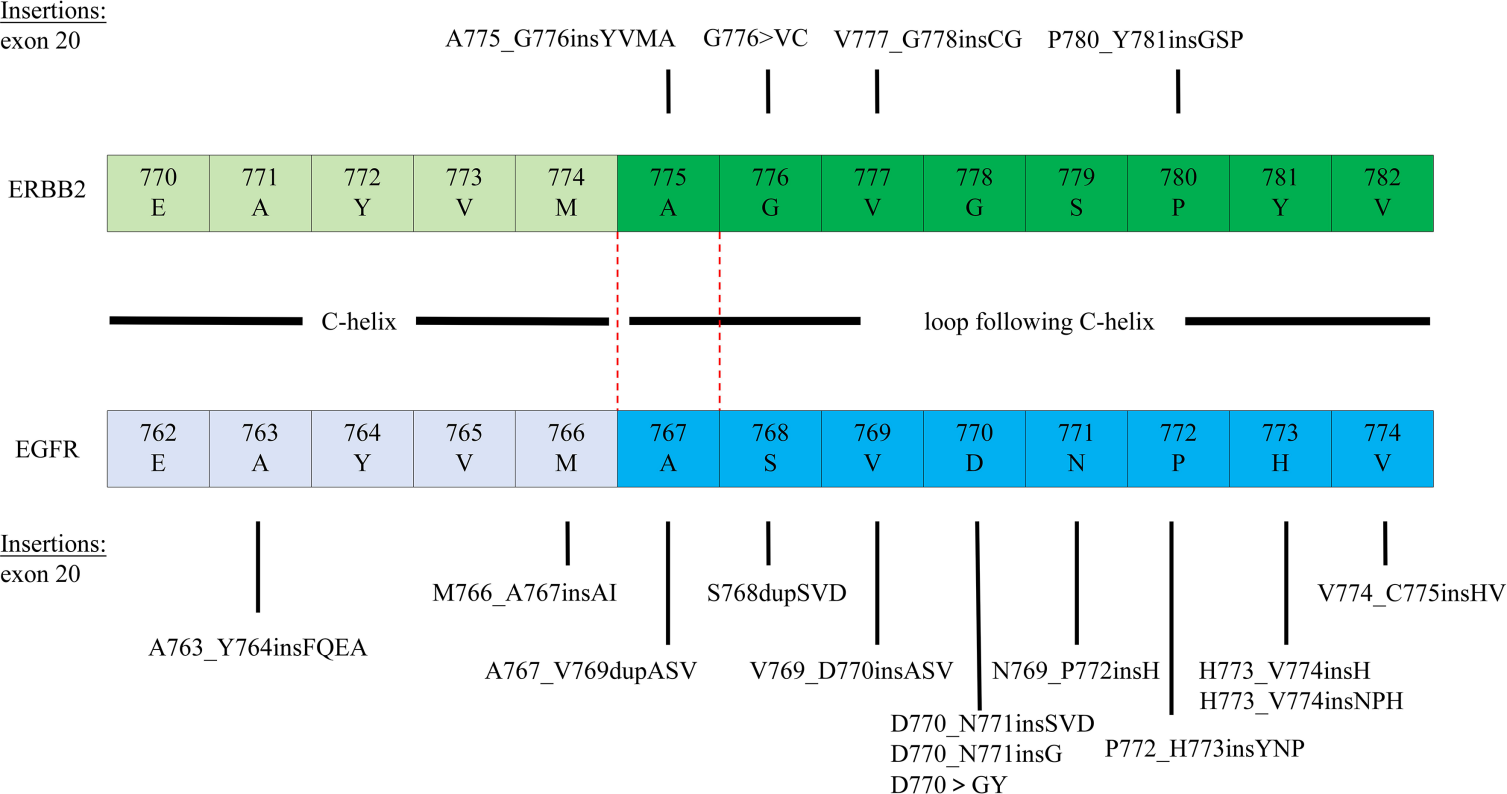
Structural homologs: EGFR and ERBB2 Exon 20 Kinase Domain Mutations. Red dashed line: highlighting the homologous mutations A767_V769dupASV (EGFR) and A775_G776insYVMA (ERBB2).

Furmonertinib is a novel, third-generation EGFR TKI with central nervous system anti-tumor activity, initially developed in China. Studies have shown that furmonertinib also elicits good responses in EGFR ex20ins mutations (7–9). Its small molecule and unique hydrophobic group form strong interactions with the L792/M793 region of the ATP-binding pocket, enabling the drug to inhibit the kinase activity of EGFR ex20ins mutations (15). Currently, there are no ongoing clinical trials investigating the efficacy and safety of furmonertinib in patients with HER2-mutant NSCLC. However, preclinical data provide a mechanistic basis for its potential activity. Studies in cell lines and murine xenograft models have demonstrated that furmonertinib and its active metabolite, AST5902, exhibit preclinical activity against HER2 exon 20 insertion mutations (e.g., V777_G778insGC[IC50 = 25nM]) and show dose-dependent antitumor efficacy (16). This preclinical evidence supports the biological plausibility of the clinical response observed in our patient. A prior case documented a response to furmonertinib in a patient with a HER2 exon 21 mutation after failure of standard therapy (17). Notably, our case is distinct in two key aspects: it involves a treatment-naïve patient and a different mutation subtype (HER2 exon 20 insertion, ERBB2 p.Y772_A775dup), representing the first report of furmonertinib’s activity in this specific frontline setting. This contrast further extends to other contexts, such as the reported activity of furmonertinib in NSCLC with co-occurring EGFR L858R and ERBB2 S310F mutations (18), which likewise differs from our case in both the specific HER2 alteration and the mutational context. Together, these observations underscore that the activity of furmonertinib may not be confined to classical EGFR mutations and may vary significantly based on the specific HER2 alteration and treatment line, highlighting the need for further genotype-directed clinical evaluation. In this case, the potential reason for this activity may lie in the shared sequence homology within the tyrosine kinase domain (TKD) between HER2 and EGFR ex20inss (**Figure 4**). The HER2 p.Y772_A775dup mutation reported here is the most common HER2 ex20ins mutation. Its insertion site is structurally homologous to the “near-loop” insertions in EGFR exon 20 (e.g., A767_V769dup) (19). Furmonertinib might “squeeze into” the deformed ATP-binding pocket caused by the mutation, thereby blocking tumor progression. However, the lack of covalent binding capability might explain potential suboptimal long-term efficacy and the need for higher doses of furmonertinib to achieve an effect. Rapid symptom relief (5-day) and disease control in our rechallenge scenario position furmonertinib as a viable alternative. The limitations of our case include a single-patient experience and incomplete long-term follow-up after treatment discontinuation. The patient’s decision to stop therapy due to financial constraints and decline further interventions limits our ability to assess progression-free survival or long-term tolerability. Nonetheless, the observed response supports further investigation of furmonertinib in HER2 ex20ins mutation NSCLC.

### Patient perspective informed consent

I find this treatment a better fit than chemotherapy. It’s tolerable, the results are encouraging, but the cost is financially challenging.

## Conclusion

This is the first report demonstrating the clinical efficacy of furmonertinib in NSCLC harboring HER2 ex20ins mutations. Furmonertinib may serve as a viable alternative for patients intolerant to chemotherapy or limited by high costs of other treatments. Further prospective studies are needed to validate its efficacy in this setting.

## Data Availability

The raw data supporting the conclusions of this article will be made available by the authors, without undue reservation.

## Glossary

HER2: human epidermal growth factor receptor 2
NSCLC: non-small cell lung cancer
T-DXd: trastuzumab deruxtecan
T-DM1: ado-trastuzumab emtansine
TKIs: tyrosine kinase inhibitors
ex20ins: exon 20 insertions
ADCs: antibody-drug conjugates
PS: performance status
HFNC: high-flow nasal cannula
PFS: progression-free survival
NCCN: National Comprehensive Cancer Network
TKD: tyrosine kinase domain

## References

[1] Siegel RL Giaquinto AN Jemal A . Cancer statistics, 2024. CA Cancer J Clin. (2024) 74:12–49. doi: 10.3322/caac.21820, PMID: 38230766

[2] Vathiotis IA Charpidou A Gavrielatou N Syrigos KN . HER2 aberrations in non-small cell lung cancer: from pathophysiology to targeted therapy. Pharm (Basel). (2021) 14:1300. doi: 10.3390/ph14121300, PMID: 34959700 PMC8705364

[3] Trillo Aliaga P Spitaleri G Attili I Corvaja C Battaiotto E Angelopoulos PA . HER2 in non-small cell lung cancer (NSCLC): evolution of the therapeutic landscape and emerging drugs-A long way to the top. Molecules. (2025) 30:2645. doi: 10.3390/molecules30122645, PMID: 40572608 PMC12195848

[4] Mazieres J Peters S Lepage B Cortot AB Barlesi F Beau-Faller M . Lung cancer that harbors an HER2 mutation: epidemiologic characteristics and therapeutic perspectives. J Clin Oncol. (2013) 31:1997–2003. doi: 10.1200/JCO.2012.45.6095, PMID: 23610105

[5] Brazel D Kroening G Nagasaka M . Non-small cell lung cancer with EGFR or HER2 exon 20 insertion mutations: diagnosis and treatment options. BioDrugs. (2022) 36:717–29. doi: 10.1007/s40259-022-00556-4, PMID: 36255589 PMC9649507

[6] Wu CP Li YC Murakami M Hsiao SH Lee YC Huang YH . Furmonertinib, a third-generation EGFR tyrosine kinase inhibitor, overcomes multidrug resistance through inhibiting ABCB1 and ABCG2 in cancer cells. Int J Mol Sci. (2023) 24:13972. doi: 10.3390/ijms241813972, PMID: 37762275 PMC10531071

[7] Yang S Liu Y Zhao J He Z Chen H Ma S . EGFR exon 20 insertions mutation in lung adenocarcinoma and its response by high-dose of Furmonertinib: a real-world study. BMC Cancer. (2025) 25:900. doi: 10.1186/s12885-025-14313-7, PMID: 40394562 PMC12090673

[8] Hu S Ming H He Q Ding M Ding H Li C . A study of high dose furmonertinib in EGFR exon 20 insertion mutation-positive advanced non-small cell lung cancer. Front Oncol. (2024) 14:1314301. doi: 10.3389/fonc.2024.1314301, PMID: 38651148 PMC11033419

[9] Zhang SS Ou SI . Spotlight on furmonertinib (Alflutinib, AST2818). The Swiss army knife (del19, L858R, T790M, exon 20 insertions, “uncommon-G719X, S768I, L861Q”) among the third-generation EGFR TKIs? Lung Cancer (Auckl). (2022) 13:67–73. doi: 10.2147/LCTT.S385437, PMID: 36317157 PMC9617553

[10] Wu R Yuan B Li C Wang Z Song Y Liu H . A narrative review of advances in treatment and survival prognosis of HER2-positive Malignant lung cancers. J Thorac Dis. (2021) 13:3708–20. doi: 10.21037/jtd-20-3265, PMID: 34277062 PMC8264687

[11] Kato Y Udagawa H Matsumoto S Izumi H Ohe Y Kato T . Efficacy of immune checkpoint inhibitors plus platinum-based chemotherapy as 1st line treatment for patients with non-small cell lung cancer harboring HER2 mutations: Results from LC-SCRUM-Asia. Lung Cancer. (2024) 197:107992. doi: 10.1016/j.lungcan.2024.107992, PMID: 39423763

[12] Mazieres J Barlesi F Filleron T Besse B Monnet I Beau-Faller M . Lung cancer patients with HER2 mutations treated with chemotherapy and HER2-targeted drugs: results from the European EUHER2 cohort. Ann Oncol. (2016) 27:281–6. doi: 10.1093/annonc/mdv573, PMID: 26598547

[13] Li BT Smit EF Goto Y Nakagawa K Udagawa H Mazieres J . Trastuzumab deruxtecan in HER2-mutant non-small-cell lung cancer. N Engl J Med. (2022) 386:241–51. doi: 10.1056/NEJMoa2112431, PMID: 34534430 PMC9066448

[14] Mazieres J Lafitte C Ricordel C Greillier L Negre E Zalcman G . Combination of trastuzumab, pertuzumab, and docetaxel in patients with advanced non-small-cell lung cancer harboring HER2 mutations: results from the IFCT-1703 R2D2 trial. J Clin Oncol. (2022) 40:719–28. doi: 10.1200/JCO.21.01455, PMID: 35073148

[15] Man X Sun X Chen C Xiang Y Zhang J Yang L . The current landscape, advancements, and prospects in the treatment of patients with EGFR exon 20 insertion mutations warrant scientific elucidation. Front Oncol. (2024) 14:1367204. doi: 10.3389/fonc.2024.1367204, PMID: 38919530 PMC11196869

[16] Musib L Kowanetz M Li Q Luo H Hu J Lutzker S . PP01.11 furmonertinib is an oral, irreversible, highly brain-penetrant pan-EGFR inhibitor with activity against classical and atypical EGFR mutations. J Thorac Oncol. (2023) 18:e14–e5. doi: 10.1016/j.jtho.2022.09.037, PMID: 41727822

[17] Ni C Zhang L Yu X Pang Y Xu J . Response to furmonertinib in a patient with non-small cell lung cancer harboring HER2 exon 21 insertion mutation: a case report. Front Oncol. (2024) 14:1440379. doi: 10.3389/fonc.2024.1440379, PMID: 39529833 PMC11551043

[18] Liu S Cao R Li H Zhang D . Furmonertinib activity in NSCLC Harbouring EGFR L858R and ERBB2 S310F co-mutations: a case report with literature review. J Chemother. (2025), 1–8. doi: 10.1080/1120009X.2025.2580760, PMID: 41185134

[19] Sentana-Lledo D Academia E Viray H Rangachari D Kobayashi SS VanderLaan PA . EGFR exon 20 insertion mutations and ERBB2 mutations in lung cancer: a narrative review on approved targeted therapies from oral kinase inhibitors to antibody-drug conjugates. Transl Lung Cancer Res. (2023) 12:1590–610. doi: 10.21037/tlcr-23-98, PMID: 37577308 PMC10413034

